# Idiopathic Isolated Mesenteric Panniculitis: A Case Report of a Rare Adipose Tissue Disease

**DOI:** 10.7759/cureus.53776

**Published:** 2024-02-07

**Authors:** Dina Z Al Shaltouni, Bassem Abou Hussein, Batool A Sawan, Leen O Saleh, Labib S Alozaibi

**Affiliations:** 1 General Surgery, Rashid Hospital / Dubai Medical College, Dubai, ARE; 2 General Surgery, Rashid Hospital, Dubai, ARE

**Keywords:** laparoscopy, adipose tissue, mesentery, abdominal pain, panniculitis

## Abstract

Mesenteric panniculitis belongs to a spectrum of rare diseases affecting the fatty tissue of the mesentery. It is characterized by chronic inflammation and fibrosis of the mesenteric adipose tissue of the bowel. Patients typically present with symptoms such as abdominal pain, nausea, vomiting, anorexia, bloating, and weight loss. Computed tomography (CT) is commonly used for diagnosis in most cases. We present a case of a 42-year-old male who experienced a significant escalation of abdominal pain over a 24-hour period. Despite seeking medical care at multiple hospitals and being prescribed analgesics, his pain remained unrelieved. Based on CT findings and the worsening pain, mesenteric panniculitis was suspected, leading to a diagnostic laparoscopy that confirmed the diagnosis. The patient was treated for idiopathic isolated mesenteric panniculitis during his hospital stay and was subsequently discharged. This article emphasizes the importance of considering mesenteric panniculitis as a possible differential diagnosis in patients with nonspecific abdominal pain, to avoid overlooking this condition.

## Introduction

Mesenteric panniculitis, also known as sclerosing mesenteritis, is among the rare diseases of the fatty tissue of the mesentery characterized by fat degeneration with necrosis and chronic inflammation [1]. It manifests with a wide range of clinical symptoms, showing considerable variability among individuals. While some may experience minimal or no noticeable symptoms, others may be significantly affected by a diverse array of complaints. These can include abdominal pain or a palpable abdominal mass, nausea, vomiting, bloating, early satiety, loss of appetite, and alterations in bowel habits such as diarrhea or constipation [2]. Patients with mesenteric panniculitis may also frequently exhibit systemic symptoms, with fever and fatigue being common complaints [3].

Diagnostic imaging techniques, such as abdominal computed tomography (CT) scans, play a crucial role in identifying the disease by revealing thickening of the mesentery and potential enlargement of lymph nodes [1]. The management of mesenteric panniculitis typically involves a multidisciplinary approach and focuses on relieving symptoms, monitoring the condition, and addressing any possible underlying causes or complications. The treatment plan varies depending on the severity of symptoms and patient factors.

## Case presentation

A 42-year-old previously healthy male presented with sudden-onset epigastric pain that started two days ago, increased in severity, worsened after meals, and was associated with anorexia. The patient conveyed their pain as abdominal tightness and rated the intensity as 8 out of 10. He denied fever, vomiting, or changes in bowel habits. He has no personal history of autoimmune disease and cancers, nor is there a family genetic predisposition. His past medical and surgical history is only significant for an open appendectomy done 25 years ago. The patient sought medical advice at a private hospital and was prescribed diclofenac (100 mg/PRN) and pantoprazole (40 mg/ BID), but they provided no relief for his symptoms after three days of usage as he claimed. The patient was afebrile and vitally stable (blood pressure: 112/77 mmHg, pulse: 65 bpm). On examination, his abdomen was soft and non-distended, with no guarding or rigidity, but moderate tenderness was noted on deep palpation of the epigastric and umbilical areas.

Initial laboratory investigations are mentioned in Table 1.

**Table 1:** Initial laboratory workup WBC: white blood count; CRP: C-reactive protein; ALT: alanine aminotransferase; AST: aspartate aminotransferase

Test	Results	Reference range
WBC (10^9^/L)	9.4	3.6-11.0
Hemoglobin (g/dL)	14.8	12.0-15.0
Hematocrit %	44.5	36.0-46.0
Platelets (10^3^/uL)	266	150-410
CRP (mg/L)	40.6	< 0.3
Lipase (U/L)	37	13 – 60
ALT (U/L)	20	0-41
AST (U/L)	13	0-37
D-dimer (μg/mL FEU)	1.8	<0.5
Lactic acid (mmol/L)	0.7	0.5 – 2.2

**Figure 1:**
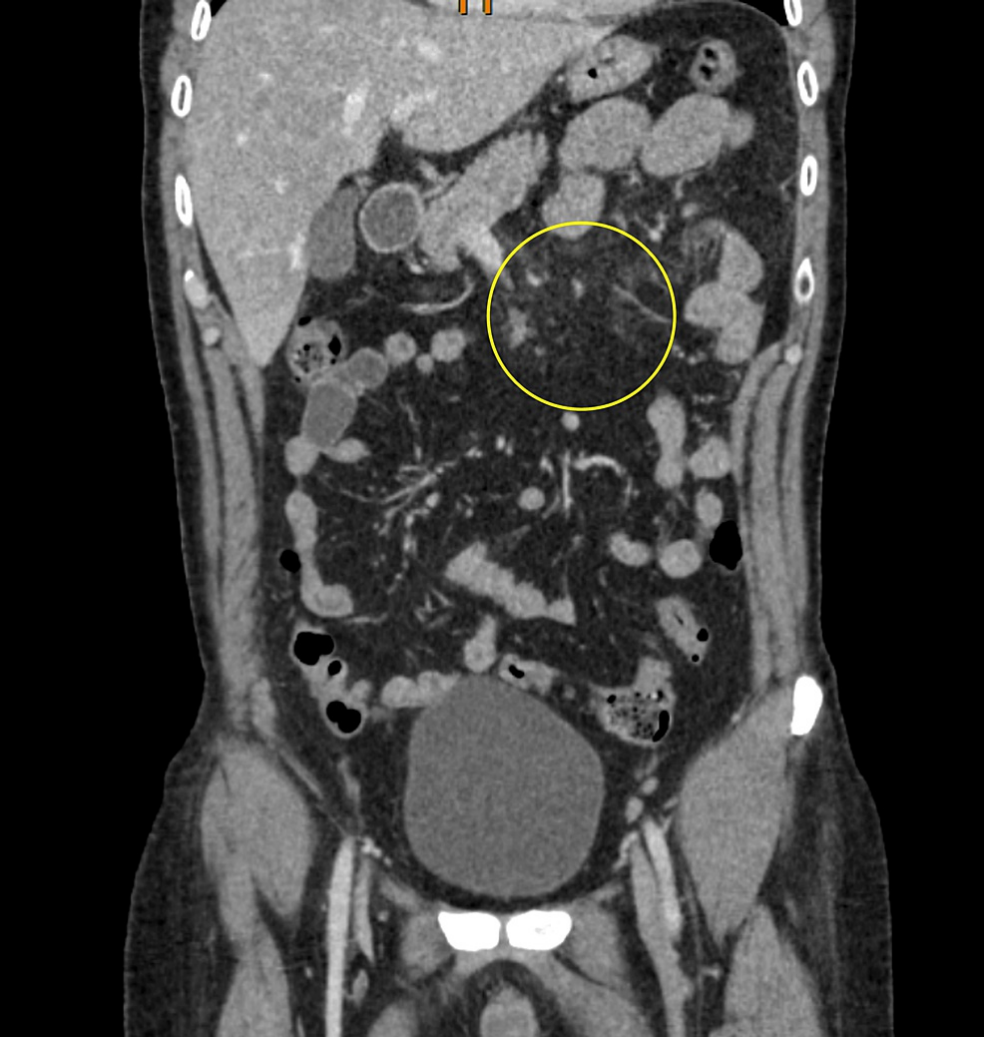
Coronal computed tomography of the abdomen with intravenous contrast The yellow circle shows mild haziness of the small bowel mesentery (misty mesentery) seen at the left hypochondrium.

A CT scan of the abdomen and pelvis was done and showed mild haziness of the small bowel mesentery, likely due to mild mesenteric panniculitis. Mesenteric ischemia was less likely, as there were no signs of bowel thickening, no presence of free fluid in the abdomen, and normal lactic acid levels in the blood. Moreover, there was no enlargement of mesenteric lymph nodes; this, coupled with the absence of gastroenteritis symptoms, excluded mesenteric lymphadenitis. Hence, mesenteric panniculitis was the likely cause of his symptoms, as there was no other significant detectable abnormality in the study (Figure 1).

**Figure 2:**
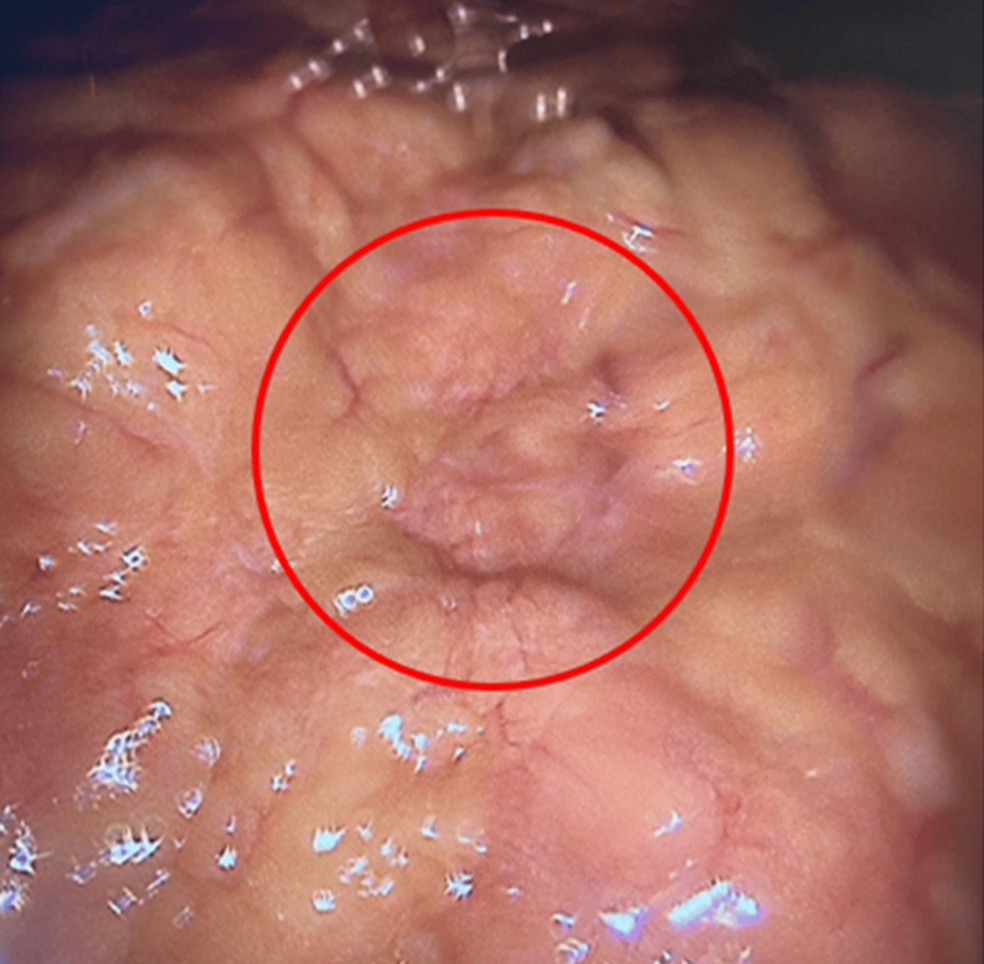
The red circle encloses ugly amalgamated fat with mild erythema and congestion

Despite receiving IV fluids and analgesics (paracetamol 500 mg 2 tablets, diclofenac 75 mg IV BID) for two days, the patient continued to complain of pain, for which a diagnostic laparoscopy was performed. During the laparoscopy, mild erythema and congestion of the mesentery were noted (Figure 2). Additionally, adhesions were found in the right lower quadrant, along with mild serous collections observed in the pelvis. No other significant abnormalities in the small and large bowels were identified. The adhesions were cut, and a sample of the serous fluid was collected and sent for cytology, which later revealed no malignant cells. No biopsies were taken because the diagnosis was relatively grossly clear during laparoscopy.

Postoperatively, the patient was stable and doing well, had minimal pain, and tolerated an oral diet. He was discharged on oral analgesics with a follow-up appointment in the General Surgery clinic. During the follow-up appointment a week later, the patient reported feeling well, with all symptoms having subsided. No complications were observed, and the pain resolved completely.

## Discussion

Mesenteric panniculitis is a relatively rare medical condition characterized by chronic, non-specific inflammation of the adipose tissue of the intestinal mesentery. It has been described as "rare," with a reported prevalence of between 0.16% and 3.4%, and a male-to-female predominance of 2 to 3:1 [4]. In over 90% of cases, mesenteric panniculitis involves the small-bowel mesentery, a rare occurrence in the colon mesentery [5]. It has often been associated with infection, abdominal surgery, vasculitis, pancreatitis, cancers, and other sclerosing disorders like Crohn's disease [3]. However, there is currently limited understanding of the pathophysiology behind the development of mesenteric panniculitis [6], especially in the absence of the mentioned causes like our case, with an autoimmune theory being the most acceptable explanation.

The clinical presentation varies, ranging from asymptomatic incidental findings on CT to abdominal pain, nausea, vomiting, anorexia, bloating, weight loss, and a palpable abdominal mass. Laboratory findings are usually nonspecific but include elevation in erythrocyte sedimentation rate, neutrophilia, and anemia. Diagnosing mesenteric panniculitis can be challenging due to its broad range of differential diagnoses, which includes various malignancies such as lymphoma, lung cancer, colon cancer, renal cell cancer, gastric carcinoma, chronic lymphocytic leukemia, Hodgkin's disease, large cell lymphoma, carcinoid tumor, and thoracic mesothelioma [2].

However, certain CT findings are considered specific to this disorder. An abdominal CT scan is the most sensitive imaging modality for detecting mesenteric panniculitis [2]. Typical signs include mesenteric thickening, fat necrosis, fibrosis, and the characteristic "fat halo sign" [7], which indicates the preservation of fat around the mesenteric vessels. Additionally, the absence of invasion into adjacent small-bowel loops, even if they are displaced, further supports the diagnosis of mesenteric panniculitis [2]. A retrospective study conducted in Turkey on patients diagnosed with mesenteric panniculitis between January 2010 and March 2016 revealed that most patients exhibited diagnostic CT findings of misty mesentery [8]. Nevertheless, the definitive diagnosis is established through a surgical biopsy performed during a laparoscopy or laparotomy, followed by histologic examination.

Management depends on the severity of the condition, with mild cases being self-limited and mostly resolving spontaneously. Treatment approaches in the literature mostly consist of supportive procedures regarding symptoms. Clinical symptoms can subside with agents such as corticosteroids, colchicine, cyclophosphamide, and tamoxifen without the need for surgery. There has been no large randomized controlled study evaluating the efficacy of steroids and immunosuppressive treatment, although clinical improvement was noted with these treatment regimens [8]. A 10-year experience from a single institute in Saudi Arabia, involving 40 patients with mesenteric panniculitis, reported that the mainstay of treatment is prednisolone alone or in combination with colchicine [5]. Rarely, a small number of individuals may experience serious complications, such as small bowel obstruction, which may require surgical intervention to relieve the obstruction [3].

The prognosis is generally good for most patients, and the outcome of the disease is usually benign [2]. However, it is not uncommon for symptoms to recur after initial management, which dictates the importance of follow-up in patients with mesenteric panniculitis. A clinical review conducted in Saudi Arabia, involving 40 patients with mesenteric panniculitis, reported the readmission of seven patients with recurrent symptoms. These patients were treated with a combination of prednisone and colchicine and were further followed up for up to two years. The results showed that the patients remained in good health and had no recurrence [5].

## Conclusions

Our case report suggests that mesenteric panniculitis can be one of the rare causes of non-specific abdominal pain, and its etiology is still unclear. Due to its vague gastrointestinal symptoms, common underlying pathologies must be excluded first before labeling it as idiopathic mesenteric panniculitis. In addition, because of the rarity of mesenteric panniculitis, it is often underdiagnosed or misdiagnosed. However, with the advancement and wide availability of CT and laparoscopy, the diagnosis is made possible.
